# Delayed Establishment of Gut Microbiota in Infants Delivered by Cesarean Section

**DOI:** 10.3389/fmicb.2020.02099

**Published:** 2020-09-11

**Authors:** Gyungcheon Kim, Jaewoong Bae, Mi Jin Kim, Hyeji Kwon, Gwoncheol Park, Seok-Jin Kim, Yon Ho Choe, Jisook Kim, Sook-Hyun Park, Byung-Ho Choe, Hakdong Shin, Ben Kang

**Affiliations:** ^1^R&D Institute, BioEleven Co., Ltd., Seoul, South Korea; ^2^Department of Food Science and Biotechnology, College of Life Science, Sejong University, Seoul, South Korea; ^3^Department of Pediatrics, Samsung Medical Center, Sungkyunkwan University School of Medicine, Seoul, South Korea; ^4^Department of Pediatrics, School of Medicine, Kyungpook National University, Daegu, South Korea

**Keywords:** newborn, gut microbiome establishment, delivery mode, neonate microbiome, available microbiome niche

## Abstract

The maternal vaginal microbiome is an important source for infant gut microbiome development. However, infants delivered by Cesarean section (CS) do not contact the maternal vaginal microbiome and this delivery method may perturb the early establishment and development of the gut microbiome. The aim of this study was to investigate the early gut microbiota of Korean newborns receiving the same postpartum care services for two weeks after birth by delivery mode using fecal samples collected at days 3, 7, and 14. Early gut microbiota development patterns were examined using 16S rRNA gene-based sequencing from 132 infants either born vaginally (VD, *n* = 64) or via Cesarean section (CS, *n* = 68). VD-born neonates showed increased alpha diversity in infant fecal samples collated at days 7 and 14 compared to those from day 3, while those of CS infants did not differ (*p* < 0.015). Bacterial structures of infants from both groups separated at day 7 (*p* < 0.001) and day 14 (*p* < 0.01). The bacterial structure of VD infants gradually changed over time (day 3 vs. day 7, *p* < 0.012; day 3 vs. day 14, *p* < 0.001). Day 14 samples of CS infants differed from day 3 and 7 samples (day 3 vs. day 14, *p* < 0.001). VD infant relative abundance of *Bifidobacterium* (days 7, 14), *Bacteroides* (days 7, 14), and *Lachnospiraceae* (day 7) significantly increased compared to CS infants, with a lower abundance of *Enterobacteriaceae* (found in all periods of the CS group) (LDA > 3.0). Relative abundances of *Bifidobacterium*, *Lactobacillus*, and *Staphylococcus* were significantly increased in both VD and CS groups at day 14 (LDA > 3.0). Predicted functional analysis showed that VD infants had overrepresented starch/sucrose, amino acid and nucleotide metabolism in gut microbiota with depleted lipopolysaccharide biosynthesis until day 14 compared to CS infants. This study confirmed that delivery mode is the major determinant of neonatal intestinal microbiome establishment and provides a profile of microbiota perturbations in CS infants. Our findings provide preliminary insight for establishing recovery methods to supply the specific microbes missing in CS infants.

## Introduction

The establishment and development of the early gut microbiome in infancy is important for immune maturation and metabolic programming (Lozupone et al., 2012). Delivery mode is a major determinant of the early gut microbial composition in infants. Infants delivered by Cesarean section (CS) miss contact with the maternal vaginal microbiome compared to infants born by vaginal delivery (VD), suggesting that CS delivery perturbs the early establishment and development of the infant gut microbiome (Dominguez-Bello et al., 2010). This immature gut microbiome in CS infants has been associated with adverse later-life outcomes such as immune and metabolic disorders (Renz et al., 2017; Stinson et al., 2018). Recently, several longitudinal studies have shown the impact of CS delivery on the establishment of early gut microbiome with subsequent health outcomes including the colonization of antimicrobial-resistance opportunistic pathogens in the gut (Shao et al., 2019), losing immune system priming (Wampach et al., 2018), and increased susceptibility to infectious outcomes (Reyman et al., 2019). The identification of missing microbes in CS infants during the early development of the gut microbiome will provide key information to develop recovery methods for the immature gut microbiome in CS infants.

Geographical location and postpartum care conditions can also affect the early life gut microbiome (Shin et al., 2015; Martin et al., 2016). Studies on the gut microbiome of Korean infants have been conducted with a limited number of subjects (less than 10 infants) or are missing early microbiome samples (Lee et al., 2015, 2016). In this study, we compared the composition, structure, and predicted metabolic function of microbiota in fecal samples from 132 healthy Korean neonates (64 from VD neonates; 68 from CS neonates) who received the same postpartum care services for 2 weeks after birth. We identified the microbial composition and predicted functions lost in the early gut microbiome of CS infants by comparing microbiota development in CS and VD infants. This study will provide sequential information on early gut microbiome establishment.

## Materials and Methods

### Study Design and Participants

This study was approved by the Institutional Review Board (IRB) of Kyungpook National University Chilgok Hospital (IRB no. 2017-06-009), and written informed consent was obtained from the mothers. Stool samples were obtained from healthy infants born between a gestational age of 37 to 41 weeks. Infants born from mothers who had GBS infection and chronic diseases such as diabetes, hypertensive disorders, or autoimmune disease were excluded. Infants born from mothers in whom oral antibiotics were administered during the third trimester of pregnancy were also excluded. For vaginal births, only infants born within 12 h of amniotic sac breakage were included. Stool samples from the infants were collected three times during the study period at 3, 7, and 14 days after birth. Clinical data regarding the mother’s gravidity and parity history, mother’s medical history, antibiotic use during pregnancy, mode of delivery, and infant’s gestational age, sex, and birth weight were recorded.

### Sample Collection

Newborn fecal samples were collected by nurses from diapers using sterile swabs at 3, 7, and 14 days after birth. These fresh fecal samples were immediately transferred to sterile cryogenic tubes and stored at −20°C until delivery to the laboratory. The samples were then stored at −80°C until DNA extraction.

### Genomic DNA Extraction

Total genomic DNA extraction was performed from 200 mg of stool sample using a QIAamp Fast DNA Stool Mini Kit (Qiagen, Germany) with additional bead beating following the manufacturer’s instructions. We measured the DNA concentration using a UV-vis spectrophotometer (Nanodrop 2000c, United States) and stored DNA samples at −20°C for subsequent experimentation.

### PCR Amplification of the V3-V4 Region of 16S rRNA

The newborn microbiota composition was analyzed by 16S rRNA amplicon sequencing using Illumina MiSeq (Illumina, Inc., United States). For sequencing, the V3-V4 region of the bacterial 16S rRNA gene was amplified using primer set F319 (5′-TCGTCGGCAGCGTCAGATGTGTATAAGAGACAGCCTACG GGNGGCWGCAG-3′) and R806 (5′-GTCTCGTGGGCTCG GAGATGTGTATAAGAGACAGGACTACHVGGGTATCTAAT CC-3′). DNA templates (12.5 ng/μL) were amplified using a KAPA HiFi Hotstart PCR Kit (Kapa Biosystems, United States) with 5 μM of primers. Reaction conditions were as follows: 95°C for 3 min, 25 cycles of 95°C for 30 s, 55°C for 30 s, and 72°C for 30 s, with a final extension at 72°C for 5 min. After PCR clean-up, a secondary amplification to attach Illumina Nextera barcodes was performed using i5 forward primer and i7 reverse primer. The DNA was amplified according to the manufacturer’s protocol. PCR products were purified using an Agencourt AMpure XP PCR Purification Kit (Beckman Coulter, United Kingdom) according to the manufacturer’s protocol. The purified products were quantified using a QuantiFluor^®^ ONE dsDNA System (Promega, United States). The product size and quality were evaluated on a Bioanalyzer 2100 (Agilent, United States). The pooled libraries were sequenced using an Illumina MiSeq instrument with a MiSeq v3 Reagent Kit (Illumina, Inc., United States). All amplicon sequence data and metadata have been uploaded through the EMP data portal (qiita.microbio.me/emp; Study ID: 13215).

### Infant Microbial Data Analyses

Analysis of the 16S rRNA sequences was performed using the QIIME (v.1.9.1) bioinformatics pipeline (Caporaso et al., 2010). Using qualified sequences (paired-end, Phred ≥ Q20), the operational taxonomic units (OTUs) were identified based on an open-reference picking method using 97% identity to entries in the Greengenes database (v13_8) (DeSantis et al., 2006) using UCLUST (Edgar, 2010). Samples were rarefied to 3 116 reads per sample and OTU biome information of all samples used in this study is provided in Supplementary Table S1. The chimeric sequences were removed using usearch61. Sample alpha diversity was calculated using the phylogenetic distance and the number of observed OTUs. For beta diversity comparison between groups, weighted/unweighted UniFrac distances were evaluated (Lozupone et al., 2011). Permutational multivariate analysis of variance (PERMANOVA) and Adonis were used to determine the significant differences in bacterial structures (Anderson, 2001). To test statistical differences, a non-parametric *t*-test with 10 000 Monte Carlo permutations was used. We carried out linear discriminant analysis effect size (LEfSe) analysis (Segata et al., 2011) to detect significant differences in bacterial taxonomies (LDA score > 3.0). To verify the number of OTUs detected per samples, OTU picking was performed and analyzed using QIIME2 (v.2020.2) (Bolyen et al., 2019) based on DADA2 workflow (Callahan et al., 2016).

Core microbiota were analyzed to identify the stability of the infant gut microbiota for each individual at days 3 and 7 based on the day 14 values (defined as the normalized OTUs present in all samples) (Mortensen et al., 2018). OTUs that are present in at least 90% of the samples at each group were selected as core components in each group. To determine the overlapped OTUs between sampling points, SourceTracker (v2) with a Bayesian approach (Knights et al., 2011) and FEAST (Shenhav et al., 2019) were used.

Phylogenetic investigation of communities by reconstruction of unobserved states (PICRUSt) was used to predict the metabolic function of the metagenomes from the 16S rRNA gene dataset (Langille et al., 2013) using Kyoto Encyclopedia of Genes and Genomes (KEGG) ortholog classification.

## Results

### Subjects

A total of 132 (64 VD and 68 CS) infants were included in this study. Clinical and anthropometric infant characteristics are presented in Table 1. There were no significant differences in the clinical characteristics of mothers and infants according to the modes of delivery except maternal age (VD = 30.9 ± 3.3, CS = 34.1 ± 4.5; *p* < 0.001). All neonates were mainly breastfed (>50%) for 2 weeks after birth.

**TABLE 1:** Characteristics of neonates included in this study according to delivery mode.

Variable	VD (*n* = 64)	CS (*n* = 68)	*p*-value
**Newborn inform**			
Male, n (%)	39 (60.9)	42 (61.7)	-
Female, n (%)	25 (39.1)	26 (38.3)	-
Birth weight, kg	3.2 ± 0.3	3.3 ± 0.4	0.183
Gestational age, w	39.0 ± 0.8	38.7 ± 1.0	0.019
**Maternal inform**			
Age, y	30.9 ± 3.3	34.1 ± 4.5	0.001

### Infant Fecal Microbiota Diversity and Structure

A total of 17 550 289 sequences (paired-end, Phred ≥ Q20) were obtained from infant fecal samples. The average sequence number per sample was 44 319 and these sequences were binned into 140 383 types of OTUs (Supplementary Table S1).

**FIGURE 1:**
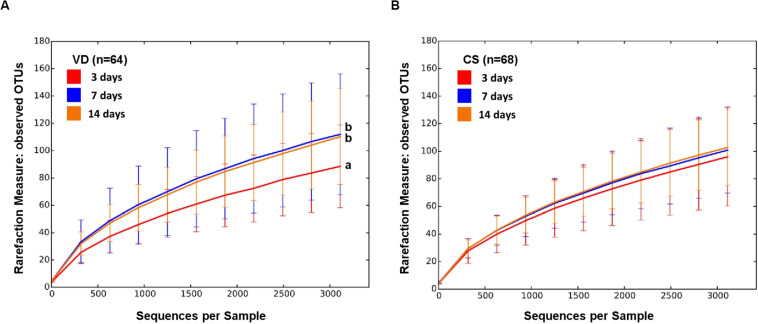
Alpha diversity of early gut microbiota according to delivery mode over time. Observed species metrics were used to plot bacterial diversity of VD **(A)** and CS **(B)**. Different letters indicate significant differences between groups (*p* < 0.05).

The alpha diversity of VD infant fecal samples increased over time but that of CS infants did not (non-parametric *t*-test, *p* < 0.015; Figure 1 and Supplementary Figure S1). While the alpha diversity of VD infant fecal samples tended to be higher in relation to CS infants, the statistical power to make comparisons was low (Supplementary Figure S1).

**FIGURE 2:**
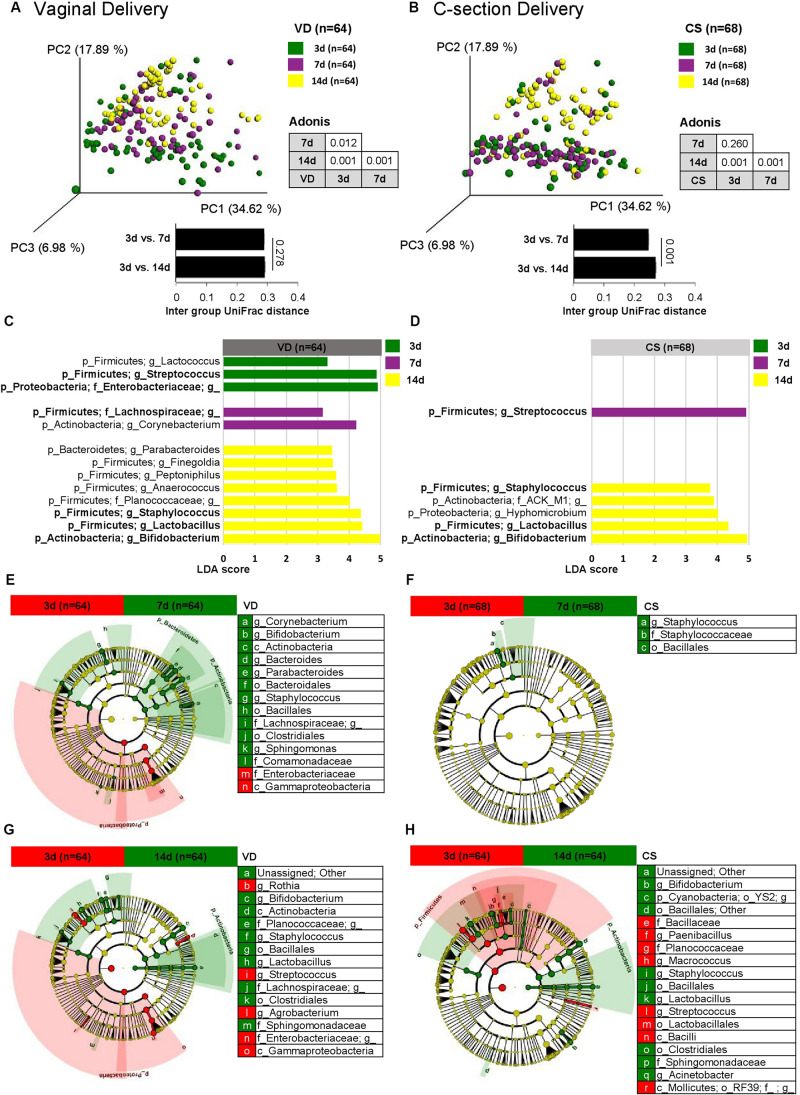
Bacterial structure differences in early gut microbiota according to delivery mode over time. Weighted UniFrac distances were used to evaluate the beta diversity of VD **(A)** and CS **(B)** infant gut microbiomes using PCoA and intra group distance. Adonis was used to test dissimilarity. LEfSe analysis (LDA > 3.0) was used to identify biomarkers between sampling points in VD **(C)** and CS infants **(D)**. Cladograms were plotted to examine the phylogenetic differences (LDA > 3.0) between sampling points in VD **(E,G)** and CS infants **(F,H)**.

Bacterial structure (beta diversity) of fecal microbiota differed significantly over time in VD infants (Figure 2A; Weighted UniFrac; Adonis, *p* < 0.012). However, the fecal microbiota of CS infants did not differ in beta diversity between day 3 and 7 samples (Figure 2B; Adonis, *p* < 0.260). This was supported by the UniFrac inter group distances between sampling days (Figures 2A,B and Supplementary Figures S2A,B).

Linear discrimination analysis (LDA) used to detect overrepresented bacterial taxonomies between sampling days showed that while six major bacterial taxonomies were overrepresented in VD infants (day 3: *Streptococcus* and *Enterobacteriaceae*; day 7: *Lachnospiraceae*; day 14: *Staphylococcus*, *Lactobacillus*, and *Bifidobacterium*), CS infants showed fewer overrepresented bacterial taxonomies (day 7: *Streptococcus*; day 14: *Staphylococcus*, *Lactobacillus*, and *Bifidobacterium*) (Figures 2C,D). Fecal microbiota on day 7 and 14 from VD infants showed a consistent profile of overrepresented bacterial taxa compared to day 3, but not in fecal samples from CS infants (Figures 2E–H).

**FIGURE 3:**
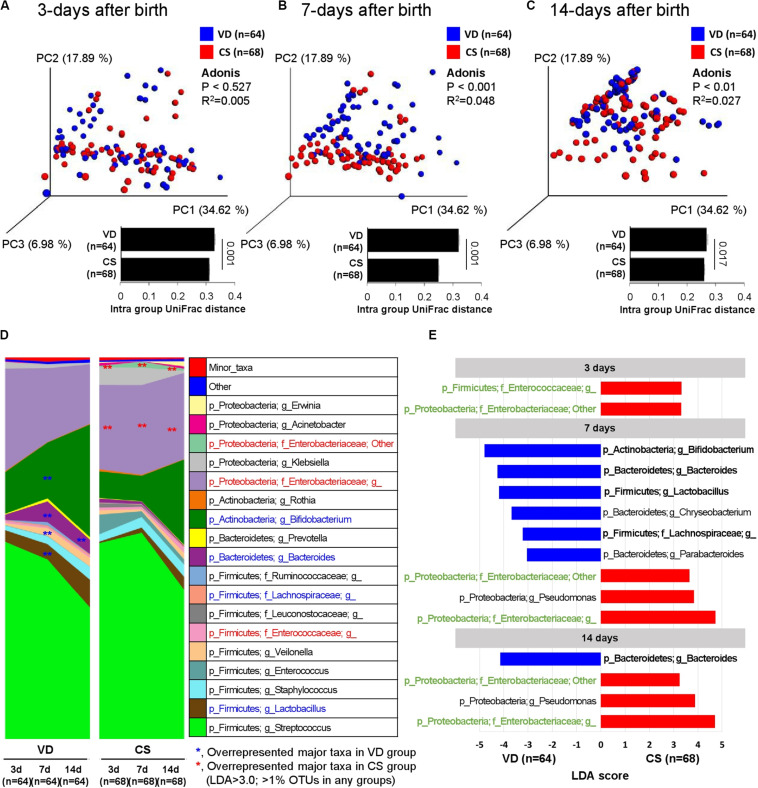
Development of early gut microbiota according to delivery mode. **(A–C)** Weighted UniFrac distances were used to evaluate beta diversity in early gut microbiota between delivery modes. Adonis was used to test dissimilarity. **(D)** Bacterial taxa plot between delivery modes. Different colors indicate each taxon at the genus level. **(E)** Differing abundance of bacterial communities between delivery modes using LEfSe; **Indicates overrepresented taxa (using LDA > 3.0) in delivery mode comparisons within sample type. Green indicates that infant gut microbiota maintenance from days 3–14 and bolded items represent the major taxa.

Fecal samples from VD infants gradually shifted to the PC2 axis on the PCoA plot over time, but CS infant fecal samples moved abruptly only after day 14, suggesting the delayed establishment of early gut microbial structure in CS infants (Figures 3A-C and Supplementary Figures S2C–G). Core microbiota ratio and source tracking results based on OTUs detected from day 3 fecal samples also supported this observation of delayed early gut microbiota establishment in CS infants (Supplementary Figure S3). *Enterococcaceae* and *Enterobacteriaceae* families were overrepresented in CS infant fecal samples over the 2 week period compared to VD infants (LDA > 3.0 of bacterial OTUs in proportions > 1%; Figures 3D,E). VD infants had a higher relative abundance of *Bifidobacterium*, *Bacteroides*, *Lactobacillus*, and *Lachnospiraceae* in fecal samples at day 7 after birth compared to CS infants (LDA > 3.0 of bacterial OTUs in proportions > 1%; Figures 3D,E). The reproducibility of dataset in this study was verified based on the latest-generation tool for sequence quality control (DADA2), demonstrated that an equivalent tendency was derived from diversity and structure of infant fecal microbiota according to the mode of delivery as the results of 97% OTU clustering methods (Supplementary Figure S4). We also could find an increased dynamic tendency in bacterial structures during the establishment of early gut microbiota (Supplementary Figure S5).

### Predictive KEGG Functional Profiling of Newborn Infant Microbiota

**FIGURE 4:**
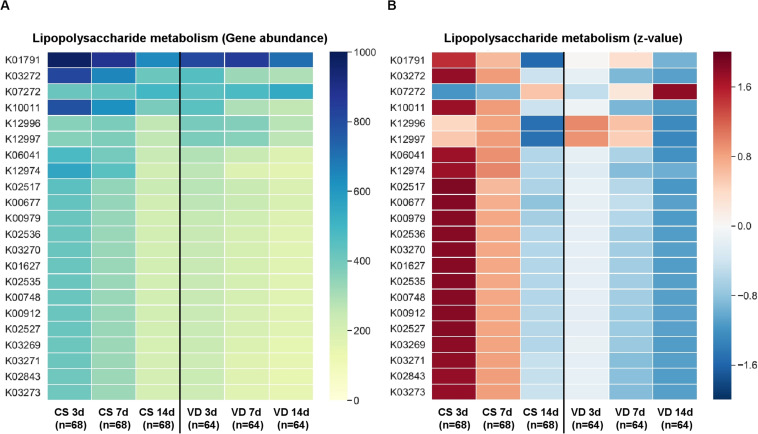
Predictive KEGG functional profiling for LPS metabolism according to delivery mode and sampling time. Gene abundance **(A)** based on PICRUSt analysis and z-score **(B)** were used to identify significant differences between groups.

PICRUSt was used to predict KEGG functional profiling from our 16s rRNA dataset. Fecal microbiota on day 7 after birth showed higher discriminant predicted functions according to delivery modes than other samples (Table 2). Fecal microbiota of VD infants at day 7 after birth had a higher proportion of bacterial genes related to starch/sucrose metabolism, pyrimidine/purine metabolism and amino acid related enzymes (LDA > 3.0), and a lower proportion of genes related to lipopolysaccharide (LPS) biosynthesis proteins (LDA > 3.0; Table 2). Various types of genes (heptosyltransferase, rhamnosyltransferase, and glycosyltransferase) related to LPS synthesis were overrepresented from day 3 and remained to day 7 after birth in CS infants (Figure 4), suggesting premature exposure to foreign microbial antigens.

**TABLE 2:** Predictive KEGG functional profiling of neonate microbiota.

KEGG functional categories		Day 3 after birth			Day 7 after birth			Day 14 after birth		
			
		VD (*n* = 64)	vs.	CS (*n* = 68)	VD (*n* = 64)	vs.	CS (*n* = 68)	VD (*n* = 64)	vs.	CS (*n* = 68)
				
Level 2	Level 3	LDA	*p* value	LDA	LDA	*p* value	LDA	LDA	*p* value	LDA
Amino acid metabolism	-	-	-	-	3.34 ←	0.00009	-	-	-	-
	Amino acid related enzymes	-	-	-	3.03 ←	0.00004	-	-	-	-
Carbohydrate metabolism	-	-	0.005	→ 3.57		0.020	→ 3.42	-	-	-
	Starch and sucrose metabolism	-	-	-	3.04 ←	0.0001	-	-	-	-
Glycan biosynthesis and metabolism	Lipopolysaccharide biosynthesis proteins	-	-	-	-	0.001	→ 3.10	-	0.002	→ 3.03
Lipid metabolism	-	-	-	-	-	0.000002	→ 3.03	-	-	-
Nucleotide metabolism	-	-	-	-	3.48 ←	0.008	-	3.29 ←	0.019	-
	Pyrimidine metabolism	-	-	-	3.21 ←	0.006	-	3.02 ←	0.015	-
	Purine metabolism	-	-	-	3.16 ←	0.014	-	-	-	-
Xenobiotics biodegradation and metabolism	-	-	-	-	-	0.0001	← 3.20	-	-	-

## Discussion

This cohort study included 132 infants that received the same postpartum care services for two weeks after birth to evaluate the development patterns of early intestinal microbiota according to delivery mode. We confirmed previous reports that delivery mode is the major determinant of neonatal intestinal microbiome establishment (Dominguez-Bello et al., 2010; Dogra et al., 2015; Mueller et al., 2016). We also identified depleted bacterial composition associated with starch/sucrose, amino acid, and nucleotide metabolism in the early gut microbiome of CS infants compared to VD infants. These intestinal microbes missing in CS infants could play an important role in postnatal immune and metabolic system development (Cox et al., 2014; Jakobsson et al., 2014). This study provides a profile of the missing microbes in infants delivered by Cesarean section and this preliminary microbial insight should be examined for its association with subsequent health outcomes.

The developmental differences of infant gut microbiota are reflected in a chronological pattern. The increased microbiota diversity observed immediately after birth may be affected by the transmission of maternal gut microbiota, including non-colonizing microbiota, in vaginally delivered infants (Lee et al., 2016). Cesarean delivery is associated with the perturbation and delayed maturation of gut microbiota in early life, which in turn has been associated with increased risk of childhood obesity (Blustein et al., 2013), asthma (Kolokotroni et al., 2012) immune diseases (Sevelsted et al., 2015), and infectious outcomes (Reyman et al., 2019). Shao et al. conducted the largest known longitudinal study including 596 infants born in United Kingdom, revealed that maternal *Bacteroides* strains are the major missing microbes in the immature gut microbiome of CS infants with colonization by hospital environment-related species including *Enterococcus*, *Enterobacter*, and *Klebsiella* (Shao et al., 2019). These modifications of early gut microbiome development by the mode of delivery is matched with our results, which showed CS infant gut microbiota enriched in *Enterobacteriaceae* and *Enterococcaceae* families with *Bacteroides* depletion.

Evaluation of the temporal pattern of the changes in intestinal microbiota diversity and composition according to the delivery mode in Korean infants during the first two weeks showed that the bacterial diversity in VD neonate stools increased from day 3 onward. However, this was not the case CS infants. These results suggested that the early gut microbiota of VD infants had an ecological niche space for early gut microbiota development. We also found that the bacterial structure of infant gut microbiota changed gradually over time in VD infants, but hierarchically (without intermediate status) in CS infants, suggesting possible constrained changes in early gut bacterial structures of CS infants. The separation in bacterial structures over time was more pronounced when the weighted UniFrac measure was used, in relation to the unweighted. These results indicated that the dominant bacterial taxa played a major role in the change of bacterial structures over time. Our findings extend the potential recovery methods for missing microbes in CS infants by specifying an appropriate environmental niche for the establishment of early gut microbiota. Dominguez-Bello et al. demonstrated that transferring the vaginal microbiome of mothers to the neonates (vaginal seeding) partially restored the microbiome of CS neonates (Dominguez-Bello et al., 2016; Mueller et al., 2019). While this procedure should be studied in a large number of well-designed trials to identify the effects on microbiome and health benefits, trials to restore the gut microbiota niche in CS neonates could minimize the risk of microbiome-related diseases. Our study excluded mothers who received antibiotics before birth, suggesting that the difference in bacterial diversity between delivery modes may depend on interactions with other factors.

In this study, the major bacterium which formed infant gastrointestinal microbiota belonged to the Firmicutes and Proteobacteria phyla (Nagpal et al., 2016; Dong et al., 2018; Nagpal and Yamashiro, 2018). Specifically, the colonization levels of several genera belonging to Firmicutes, such as *Enterococcoceae*, *Lachnospiraceae*, and *Lactobacillus* were significantly influenced by delivery mode (Gronlund et al., 1999; Rutayisire et al., 2016). Other previous studies also showed that higher *Enterobacteriaceae* and *Pseudomonas* levels were observed in CS compared to VD infants (Adlerberth et al., 2006; Dominguez-Bello et al., 2010; Hesla et al., 2014; Dogra et al., 2015). The possible sources of these overrepresented microbes in CS infants should be examined to guide postnatal conditions for the establishment of early gut microbiota (Fryklund et al., 1992; Shin et al., 2015). Biomarker analysis using LEfSe also showed a higher relative abundance of *Lactobacillus*, *Bifidobacterium*, and *Bacteroides* in the stools of VD infants compared to those of CS infants; these findings are supported by previous studies (Mitsou et al., 2008; Kabeerdoss et al., 2013). The *Bacteroides* genus is a typical adult intestinal bacterium and several studies suggest that this genus may be transmitted from the maternal gut to neonates during delivery (Adlerberth et al., 2007; Vaishampayan et al., 2010; Rutayisire et al., 2016). The delayed establishment of *Bacteroides* in the gut microbiota of CS neonates could be related to health outcomes (Adlerberth et al., 2006; Penders et al., 2006). Some *Bacteroides* species (*B. fragilis*) are known to activate T cell-dependent immune responses that can affect both the development and homeostasis of the host immune system (Troy and Kasper, 2010). The predicted functional profiles related to LPS and xenobiotic metabolism were overrepresented in the early gut microbiome of CS infants at day 7 after birth (compared to VD infants). These differences are primarily contributed by enriched *Enterobacteriaceae* populations producing several types of LPS. Several studies show that lower LPS biosynthesis is related to developing a protective effect against asthma (Lotz et al., 2006; Stiemsma and Turvey, 2017), suggesting that finely timed exposure to foreign microbial antigens may help neonate immune system development. Interrogation of these predicted functional differences needs to be confirmed in future cohorts using true shotgun metagenomics analyses or metabolomics.

This study showed that the ratio of core gut microbiota from CS infants partially recovered by day 14 after birth compared to those of VD infants. This partial restoration of early gut microbiota from CS infants occurred relatively earlier than previously reported (Palmer et al., 2007; Eggesbo et al., 2011; Liu et al., 2019). While overrepresented *Enterobacteriaceae* and depleted *Bacteroides* were maintained at day 14 in CS infants, *Bifidobacterium* and *Lactobacillus* were restored to a level similar to those of VD infants. This reveals that a strategy for restoring microbiota is needed, excluding remedial supplementation with probiotic microbes. Also, further studies using Korean infants will help to identify factors causing the relatively rapid restoration of early gut microbiota in CS infants, such as Korean postpartum services (Kyung Kim et al., 2017).

A strength of this study is geographic specificity with a large sample size and density of sampling at early time points. However, limitations of this study included a lack of maternal samples (stools, skin/vaginal swabs) and differences in maternal and gestational ages (Table 1). The previous investigation on the impact of exclusive breastfeeding on gut microbiota in CS infants showed partial restoration of early gut microbiota establishment, in relation to VD infants (Liu et al., 2019). While we only documented whether the infants were majoritarily breastfed for the type of feeding, this study was limited to screen the exclusively breastfed group. As such, we are unable to suggest a direct link between other important cofactors (maternal microbiota and breastfeeding status) and infant early gut microbiota. Additionally, other limitations are the lack of follow-up measures to identify health outcomes and the rough resolution reachable by 16S rDNA. Further well-designed studies with multi-omics approaches are needed to increase our knowledge on the impact of mother-to-newborn microbiome transfer.

## Data Availability Statement

The original contributions presented in the study are publicly available. This data can be found here: https://www.ebi.ac.uk/ena/browser/view/PRJEB39707.

## Ethics Statement

The studies involving human participants were reviewed and approved by Kyungpook National University Chilgok Hospital. Written informed consent to participate in this study was provided by the participants’ legal guardian/next of kin.

## Author Contributions

S-JK, YHC, HS, and BK conceived this study. YHC, JK, S-HP, B-HC, and BK designed the study and collected the microbiome samples. GK, JB, MJK, HK, GP, and HS analyzed the data. GK, JB, MJK, S-JK, HS, and BK wrote the manuscript. All authors contributed with valuable discussions and edition, approving the final version of manuscript.

## Conflict of Interest

GK, JB, HK, and S-JK were employed by BioEleven Co., Ltd. The remaining authors declare that the research was conducted in the absence of any commercial or financial relationships that could be construed as a potential conflict of interest.

## Supplementary Material

FIGURE S1 Alpha diversity of early gut microbiota according to delivery mode over time. **(A)** Observed species metric. **(B)** Phylogenetic diversity. Non-parametric *p*-value was calculated using 10,000 Monte Carlo permutations.

FIGURE S2 Bacterial structure differences of early gut microbiota according to delivery mode over time. Unweighted UniFrac distances were used to evaluate beta diversity of VD infants **(A)** and CS infants **(B)** using PCoA and intra group distance. Adonis was used to test dissimilarity. **(C–E)** Unweighted UniFrac distances were used to evaluate beta diversity between delivery modes. Cladograms were plotted to examine phylogenetic differences (LDA > 3.0) between sampling points in VD infants **(F)** and CS infants **(G)**.

FIGURE S3 The ratio of core microbiota differences of early gut microbiota according to delivery mode over time. The normalized OTUs present in all samples were used to calculate the ratio of core microbiota in stool samples from VD infants **(A)** and CS infants **(B)**. Source tracking analyses were performed to identify the ratio of overlapped OTUs between sampling points using Source Tracker2 **(C)** and FEAST **(D)**.

FIGURE S4 Bacterial alpha and beta diversity in early gut microbiota according to delivery mode over time based on QIIME2 pipeline. Observed species metrics were used to plot bacterial diversity of VD **(A)** and CS **(B)**. Weighted UniFrac distances were used to evaluate the beta diversity of VD **(C)** and CS **(D)** infant gut microbiomes using PCoA and intra group distance. PERMANOVA was used to test dissimilarity. **(E)** Bacterial taxa plot between delivery modes. Different colors indicate each taxon at the genus level; ^∗∗^Indicates overrepresented taxa (using LDA > 3.0) in delivery mode comparisons within sample type.

FIGURE S5 Increased dynamics of bacterial structures during the establishment of early gut microbiota. Shannon diversity metrics were used to plot bacterial diversity of all samples **(A)**, VD group **(B)**, and CS group **(C)**. Non-parametric *p*-value was calculated using 10,000 Monte Carlo permutations. Individual bacterial taxa plots at the genus level of VD group **(D)** and CS group **(E)**. Different colors indicate each taxon at the genus level.

TABLE S1 OTU biome information of all samples used in this study.
