# Supplementary material for: Delayed Establishment of Gut Microbiota in Infants Delivered by Cesarean Section
Source: Front Microbiol. 2020 Sep 11;11:2099. doi: 10.3389/fmicb.2020.02099 (PMC7516058; doi:10.3389/fmicb.2020.02099)
Supplement: FIGURE S1 — Alpha diversity of early gut microbiota according to delivery mode over time. (A) Observed species metric. (B) Phylogenetic diversity. Non-parametric p-value was calculated using 10,000 Monte Carlo permutations. [file Data_Sheet_1.DOCX]

Supplementary Material

# Supplementary Figures and Tables


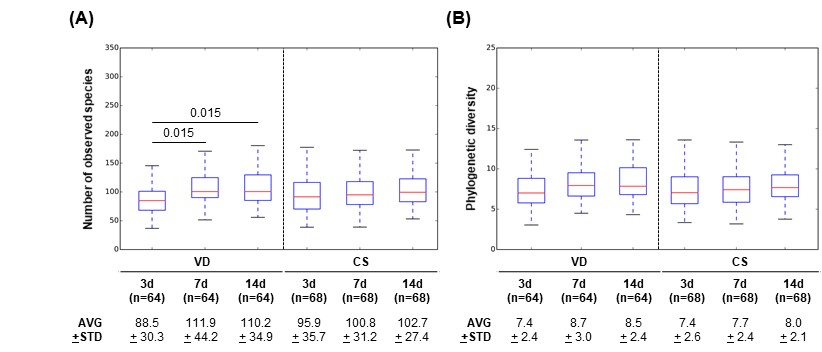


**Supplementary Figure 1**. Alpha diversity of early gut microbiota according to delivery mode over time. (A) Observed species metric. (B) Phylogenetic diversity. Non-parametric p-value was calculated using 10,000 Monte Carlo permutations.

**
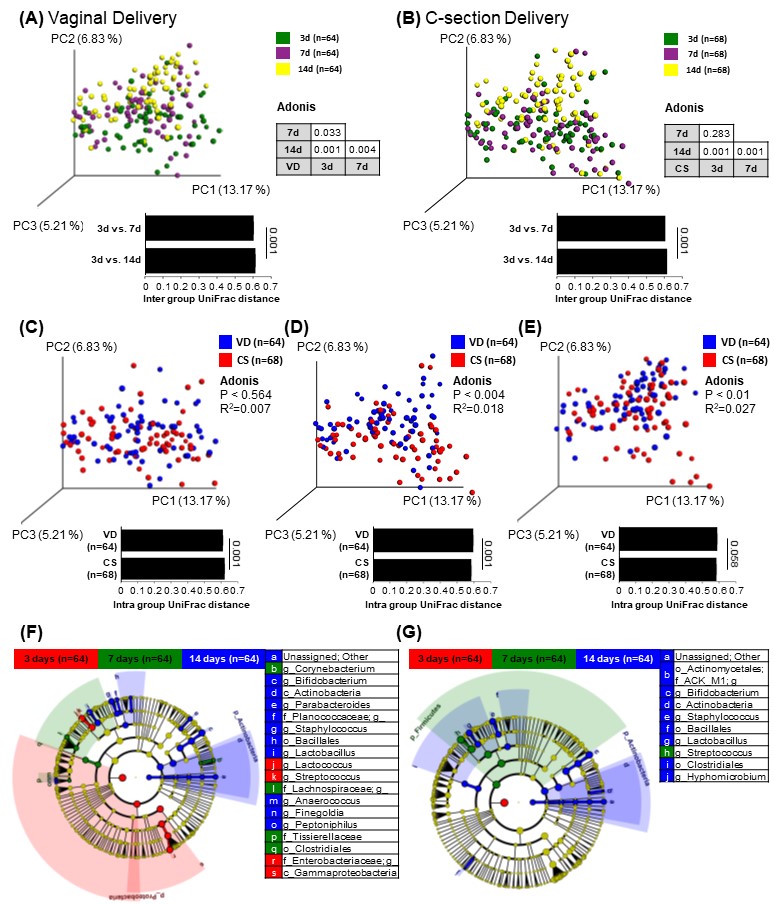
**

**Supplementary Figure 2**. Bacterial structure differences of early gut microbiota according to delivery mode over time. Unweighted UniFrac distances were used to evaluate beta diversity of VD infants (A) and CS infants (B) using PCoA and intra group distance. Adonis was used to test dissimilarity. (C-E) Unweighted UniFrac distances were used to evaluate beta diversity between delivery modes. Cladograms were plotted to examine phylogenetic differences (LDA>3.0) between sampling points in VD infants (F) and CS infants (G).

**
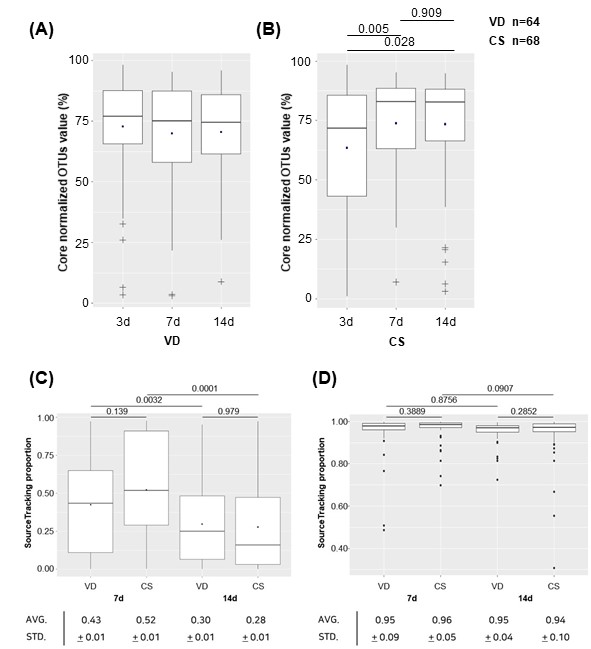
**

**Supplementary Figure 3**. The ratio of core microbiota differences of early gut microbiota according to delivery mode over time. The normalized OTUs present in all samples were used to calculate the ratio of core microbiota in stool samples from VD infants (A) and CS infants (B). Source tracking analyses were performed to identify the ratio of overlapped OTUs between sampling points using Source Tracker2 (C) and FEAST (D).


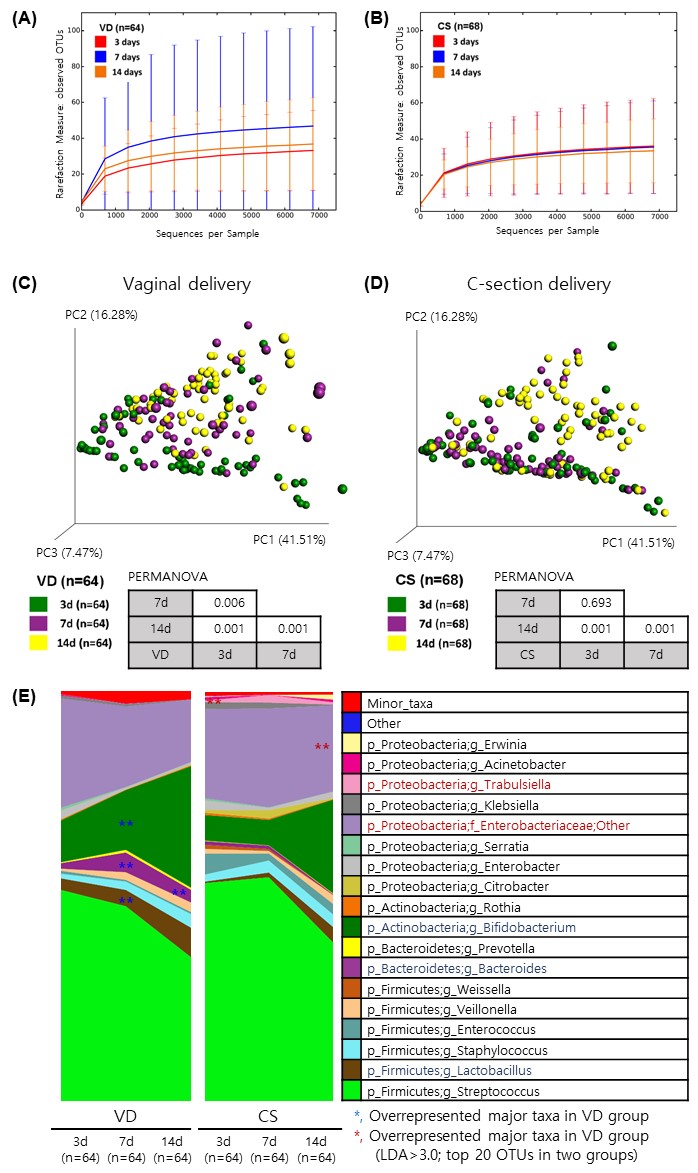


**Supplementary Figure 4**. Bacterial alpha and beta diversity in early gut microbiota according to delivery mode over time based on QIIME2 pipeline. Observed species metrics were used to plot bacterial diversity of VD (A) and CS (B). Weighted UniFrac distances were used to evaluate the beta diversity of VD (C) and CS (D) infant gut microbiomes using PCoA and intra group distance. PERMANOVA was used to test dissimilarity. (E) Bacterial taxa plot between delivery modes. Different colors indicate each taxon at the genus level; ** Indicates overrepresented taxa (using LDA > 3.0) in delivery mode comparisons within sample type.


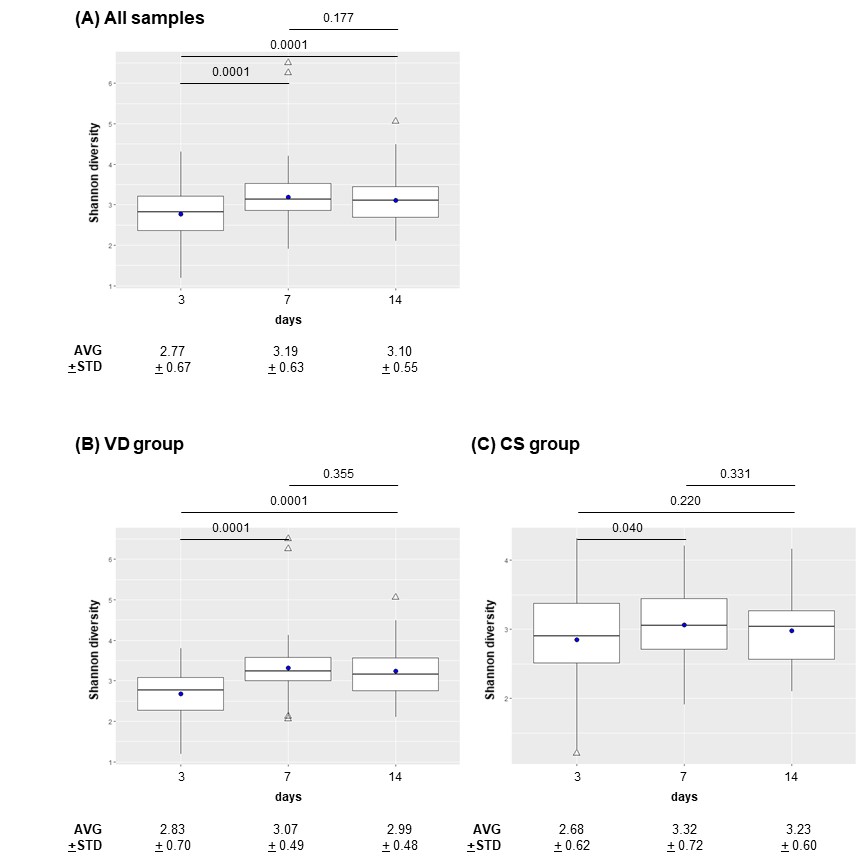


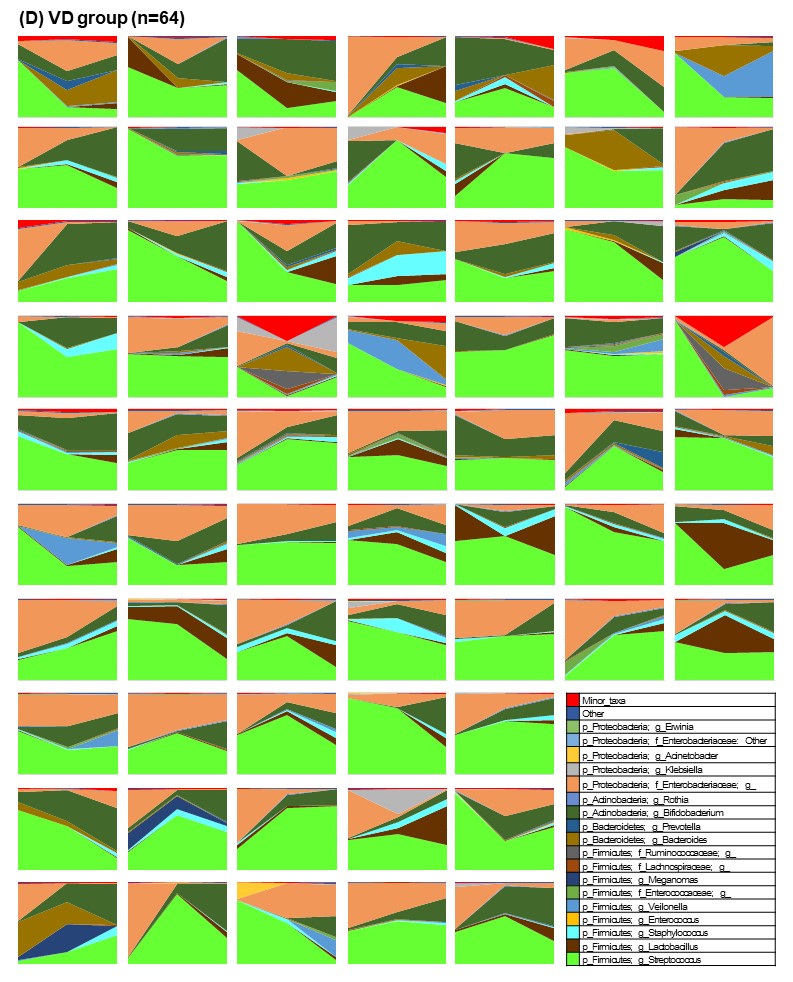


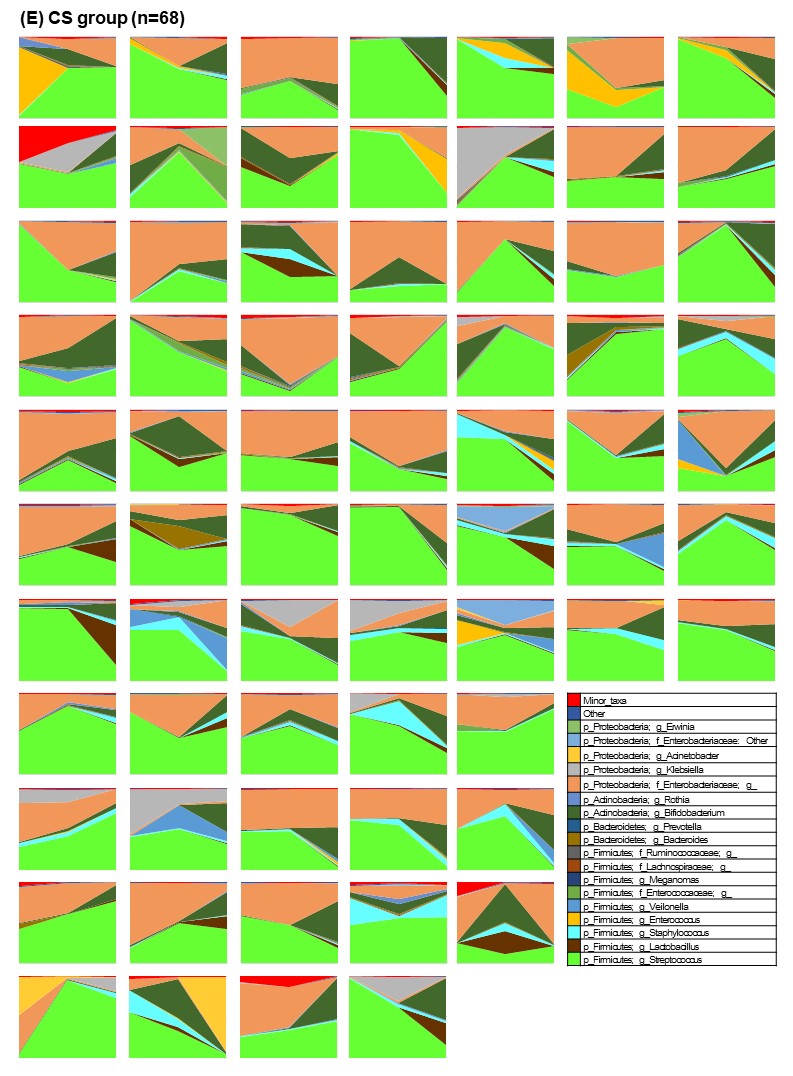


**Supplementary Figure 5**. Increased dynamics of bacterial structures during the establishment of early gut microbiota. Shannon diversity metrics were used to plot bacterial diversity of all samples (A), VD group (B), and CS group (C). Non-parametric p-value was calculated using 10,000 Monte Carlo permutations. Individual bacterial taxa plots at the genus level of VD group (D) and CS group (E). Different colors indicate each taxon at the genus level.

**
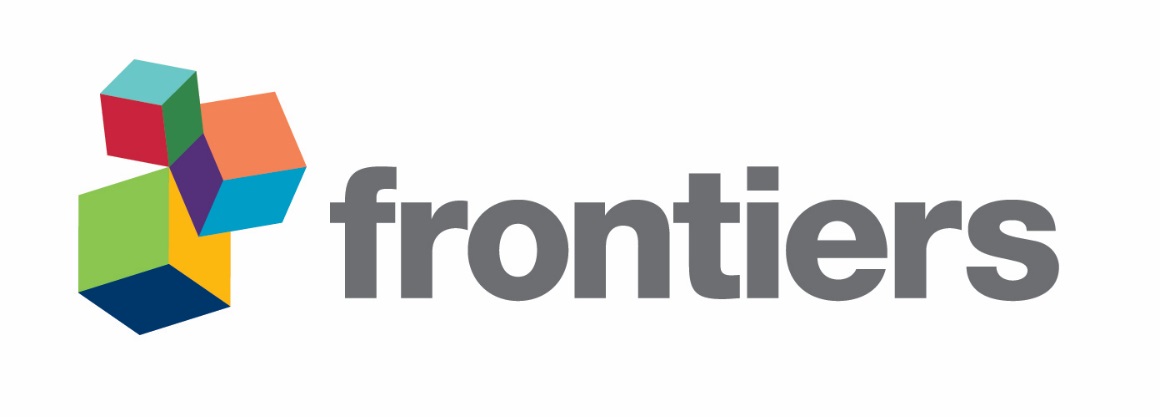
**
